# Haploinsufficiency for Translation Elongation Factor eEF1A2 in Aged Mouse Muscle and Neurons Is Compatible with Normal Function

**DOI:** 10.1371/journal.pone.0041917

**Published:** 2012-07-25

**Authors:** Lowri A. Griffiths, Jennifer Doig, Antonia M. D. Churchhouse, Faith C. J. Davies, Charlotte E. Squires, Helen J. Newbery, Catherine M. Abbott

**Affiliations:** Centre for Molecular Medicine, Institute of Genetics and Molecular Medicine, University of Edinburgh, Western General Hospital, Edinburgh, United Kingdom; National Institute of Health, United States of America

## Abstract

Translation elongation factor isoform eEF1A2 is expressed in muscle and neurons. Deletion of eEF1A2 in mice gives rise to the neurodegenerative phenotype “wasted” (*wst*). Mice homozygous for the wasted mutation die of muscle wasting and neurodegeneration at four weeks post-natal. Although the mutation is said to be recessive, aged heterozygous mice have never been examined in detail; a number of other mouse models of motor neuron degeneration have recently been shown to have similar, albeit less severe, phenotypic abnormalities in the heterozygous state. We therefore examined the effects of ageing on a cohort of heterozygous *+/wst* mice and control mice, in order to establish whether a presumed 50% reduction in eEF1A2 expression was compatible with normal function. We evaluated the grip strength assay as a way of distinguishing between wasted and wild-type mice at 3–4 weeks, and then performed the same assay in older *+/wst* and wild-type mice. We also used rotarod performance and immunohistochemistry of spinal cord sections to evaluate the phenotype of aged heterozygous mice. Heterozygous mutant mice showed no deficit in neuromuscular function or signs of spinal cord pathology, in spite of the low levels of eEF1A2.

## Introduction

The wasted mouse is a model for early onset severe motor neuron degeneration [1], [2]. The underlying mutation arose in 1972 at the Jackson lab, where it was shown to be recessive and autosomal [3]. We subsequently found that the primary lesion was a 15.8 kb deletion which removed the first exon and promoter of the gene encoding a tissue-specific translation elongation factor called eEF1A2 [4]. This isoform is closely related to eEF1A1, but whereas eEF1A1 is almost ubiquitously expressed, eEF1A2 is expressed only in neurons, cardiac and skeletal muscle [5], [6], [7]. The timing of the switch from eEF1A1 to eEF1A2 expression in post-natal neurons and muscle, which is complete by 21 days, fits perfectly with the timing of the onset of the phenotype in wasted mice; *wst/wst* homozygotes are outwardly normal until 21 days and then deteriorate and die within the next 7 days. During this time they lose muscle bulk, develop gait abnormalities and display changes in the spinal cord consistent with those seen in humans with motor neuron disease, including loss of motor neurons, perikaryal accumulation of neurofilaments and reactive gliosis [2]. Wasted mice also perform increasingly poorly on the rotarod over the same period of time.

A number of mutations in mouse genes related to motor neuron disease have been shown to have late-onset phenotypic effects in heterozygotes, in contrast to a much more severe and/or early onset phenotype in homozygotes. For example, mutations in the dynein heavy chain in mice give rise to the “legs at odd angles” phenotype, characterised by motor neuron degeneration by 16 months, whereas homozygous mutant animals die within 24 hours after birth [8]. Similarly, a targeted mutation in the *Zpr1* gene (coding for a zinc finger protein, also called ZNF259) in mice gives rise to an early embryonic lethal phenotype in homozygous *Zpr1^−/−^* mice. In contrast, heterozygotes are viable; they develop progressive motor neuron loss but not at a significant level until 12 months of age [9]. Importantly, these mice display progressive down-regulation of Zpr1 in brains; at 6 weeks there was only a 15% reduction in expression compared to wild-type tissues, but by 12 months the reduction was the expected 50%. As ZPR1 has been shown to interact with eEF1A [10] and survival motor neuron (SMN) proteins [11] (mutations of which cause spinal muscular atrophy) we decided to investigate expression levels of eEF1A2 in older wasted heterozygote (*wst*/+) mice, and to establish whether they showed any signs of late onset motor neuron degeneration.

We therefore set out to examine the effects of ageing on a cohort of heterozygous *+/wst* mice together with wild-type littermate controls, in order to establish whether a reduction in eEF1A2 expression was compatible with normal function. Although the wasted mutation was originally said to be recessive on the basis that heterozygotes survived and bred, the only pathological investigations that had been carried out previously were on *+/wst* mice of less than a month old. Similarly, no expression analysis of eEF1A2 had been carried out on wasted heterozygotes past 3 months of age.

We also set out to evaluate the use of a grip strength meter as a way of distinguishing between wasted and wild-type mice at 3–4 weeks, and then performed the same assay in older *+/wst* and wild-type mice. Finally, we used performance on the rotarod and immunohistochemistry of spinal cord sections to evaluate the phenotype of aged heterozygous mice.

**Table 1:** Motor neuron numbers from the spinal cords of the ageing cohort.

Group	Level of spinalcord	Number of mice	Mean MN count	Minimum MN count	Maximum MN count	Standard deviation
Male +/+	Cervical	6	30.3	17	45	9.4
	Thoracic	6	33.3	16	49	12.6
	Lumbar	4	26.3	19	38	8.4
Male *+/wst*	Cervical	5	32.6	29	37	3.8
	Thoracic	6	29.1	25	38	4.6
	Lumbar	4	14.4	10	32	9.6

Mean motor neuron counts from spinal cord sections from the ageing cohort. WT indicates wild-type animals, HET indicates heterozygote animals N  =  number of animals in the group, Mean  =  average of group, Min  =  minimum reading of group, max  =  maximum reading of group, S.D  =  standard deviation. No differences were significant.

## Materials and Methods

### Mice

Mice were housed in the Biomedical Research Facility (BRF) at the University of Edinburgh. All mice were maintained in accordance with Home Office regulations and all protocols had been approved by the local ethics committee of the University of Edinburgh. All animals were housed in single sex cages but each cage contained a mixture of both wild-type and heterozygous animals.

### Western Blotting

Western blotting was carried out as previously described [7] using anti-eEF1A antibodies raised in sheep. Anti-eEF1A2 was used at 1∶2000.

**Figure 1:**
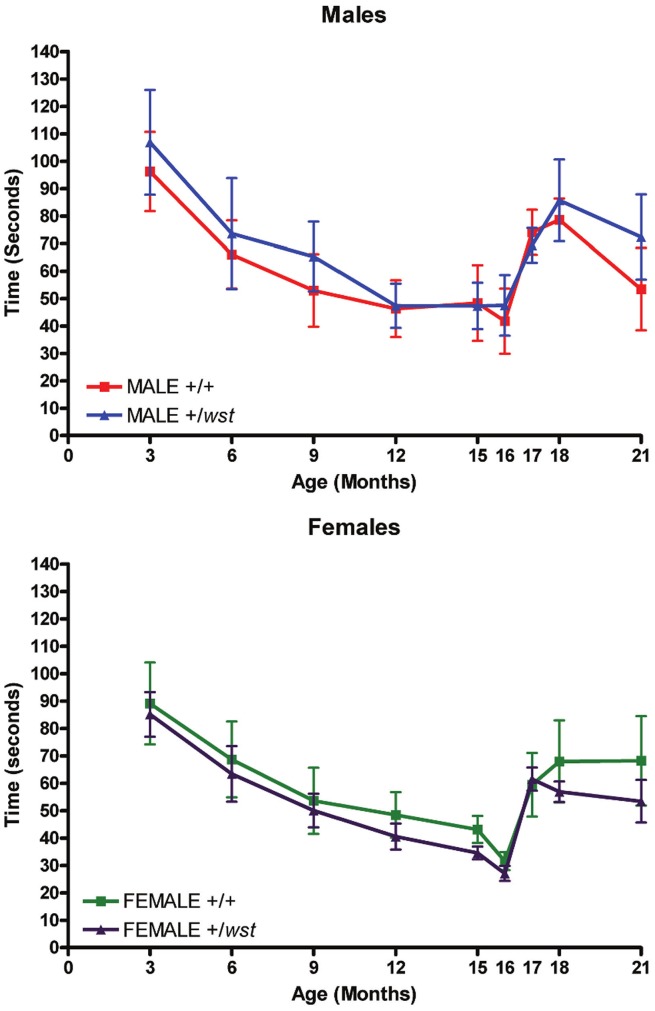
Rotarod analysis of aged mice. Mean times of latency to fall from the rotarod over the 21 months of the ageing study. WT indicates wild-type animals, HET indicates heterozygote animals. The upper graph shows data from the male groups and the lower graph shows data from the female groups. Error bars represent the standard error of the mean.

In case the repeated exposure to the rotarod was masking any difference between genotypes (because of beneficial effects of exercise, or familiarity with the task), a second cohort was tested at 18 months only, again over a 5 day period. However, there were still no statistically significant differences between genotypes (data not shown).

### Rotarod Analysis

Mice were placed on a rotating beam and their latency in falling from the beam recorded. Mice were trained for 5 minutes at a constant speed of 4 rpm, followed by a 30 minute rest period, and then tested 3 times at accelerating speed (4–40 rpm over 5 minutes) with 15 minute recovery periods in between each test. Mice were tested on alternate days over a fortnight. To ensure animals did not require extensive re-training at the start of each testing month, between testing periods mice were trained once a week for 5 minutes on the rotarod set to a constant speed of 4 rpm to maintain familiarity with the procedure. All rotarod data were collected blind. At the start of each testing session mice were monitored for several minutes and weighed to check for any obvious mobility or other health problems. Once mice were deemed healthy training commenced. If however a mouse showed any signs of illness they were immediately removed from the study and all data for that animal for that given month was excluded.

**Figure 2:**
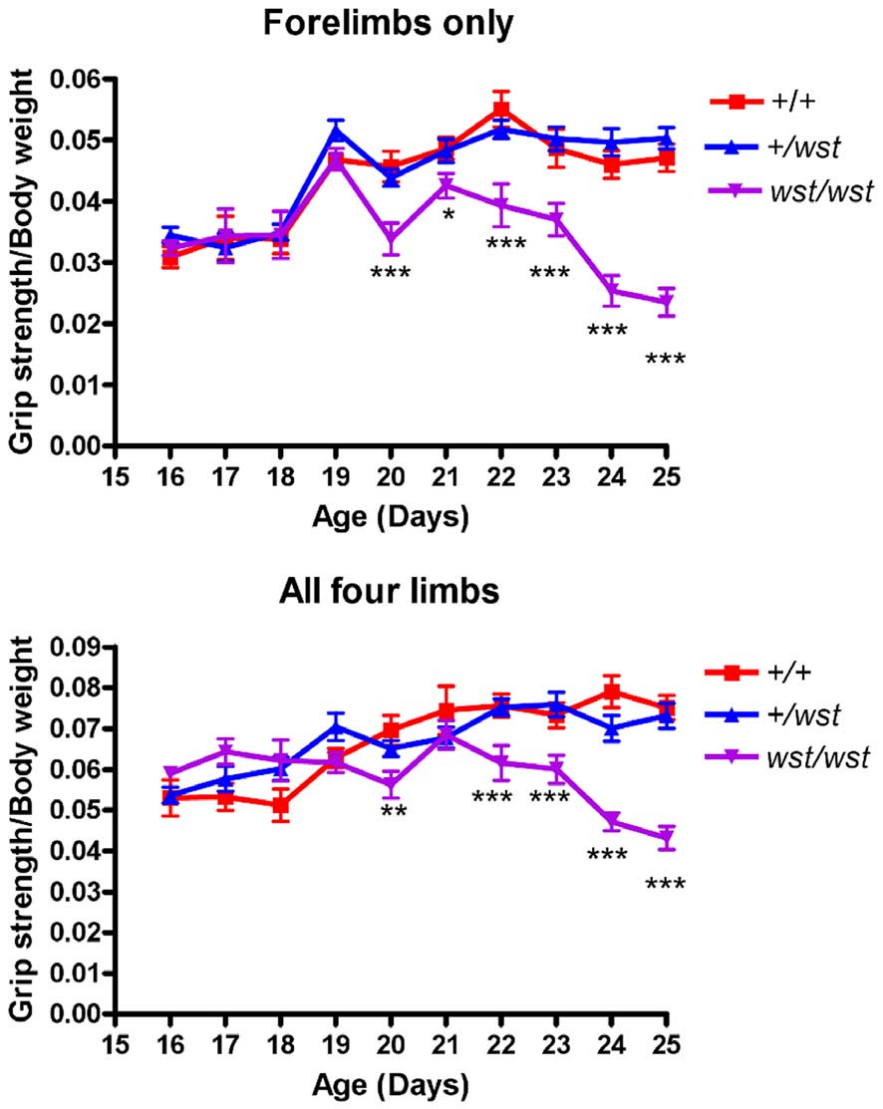
Grip strength analysis of young wasted mice. Forelimb (top panel) and all four limbs (bottom panel) grip strength analysis of wasted mice. The daily grip strength reading of 3 tests (measured in Newtons) were normalised to body weight (measured in grams). P values were calculated comparing wasted mice with controls (+/+ and *+/wst* combined). * indicates a P value<0.05, ** indicates a P value<0.01, and *** indicates a P value <0.001.

### Grip Strength Meter

A grip strength meter from Bioseb was used for this assay. Mice were tested for muscle strength in forelimbs only and then in all four limbs. To account for variation in body weight all grip strength readings (measured in Newtons) were normalised to body weight (measured in grams).

### Immunohistochemistry

Immunohistochemistry was carried out as previously described [7]. Antibodies used were eEF1A2 [7] used at 1∶10 dilution, anti-GFAP raised in rabbit (Dako) at 1∶1000 and anti-NF-H raised in mouse (Dako) and 1∶80. Spinal cord sections (4 microns) were stained with eEF1A2 and motor neurons of the anterior horn section of the spinal cord (as defined by Fischer et al [12] ) were counted. One section was counted at each level for each mouse (4–6 mice, as shown in table 1).

**Figure 3:**
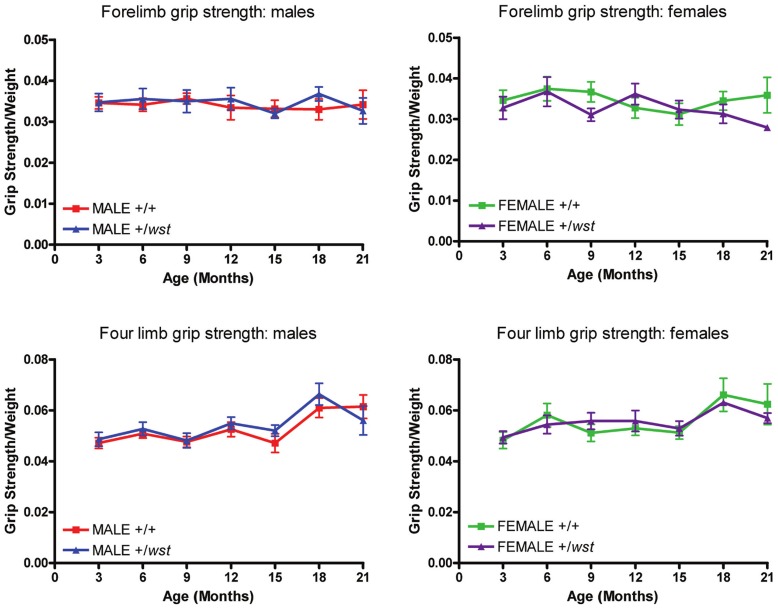
Grip strength analysis of aged mice. Grip strength data for the entire ageing study. Graphs on the left display male data and graphs on the right display female data. Top graphs display forelimb data only and the bottom 2 graphs display data from all four limbs. WT indicates wild-type animals, HET indicates heterozygote animals. Error bars represent the standard error of the mean.

**Figure 4:**
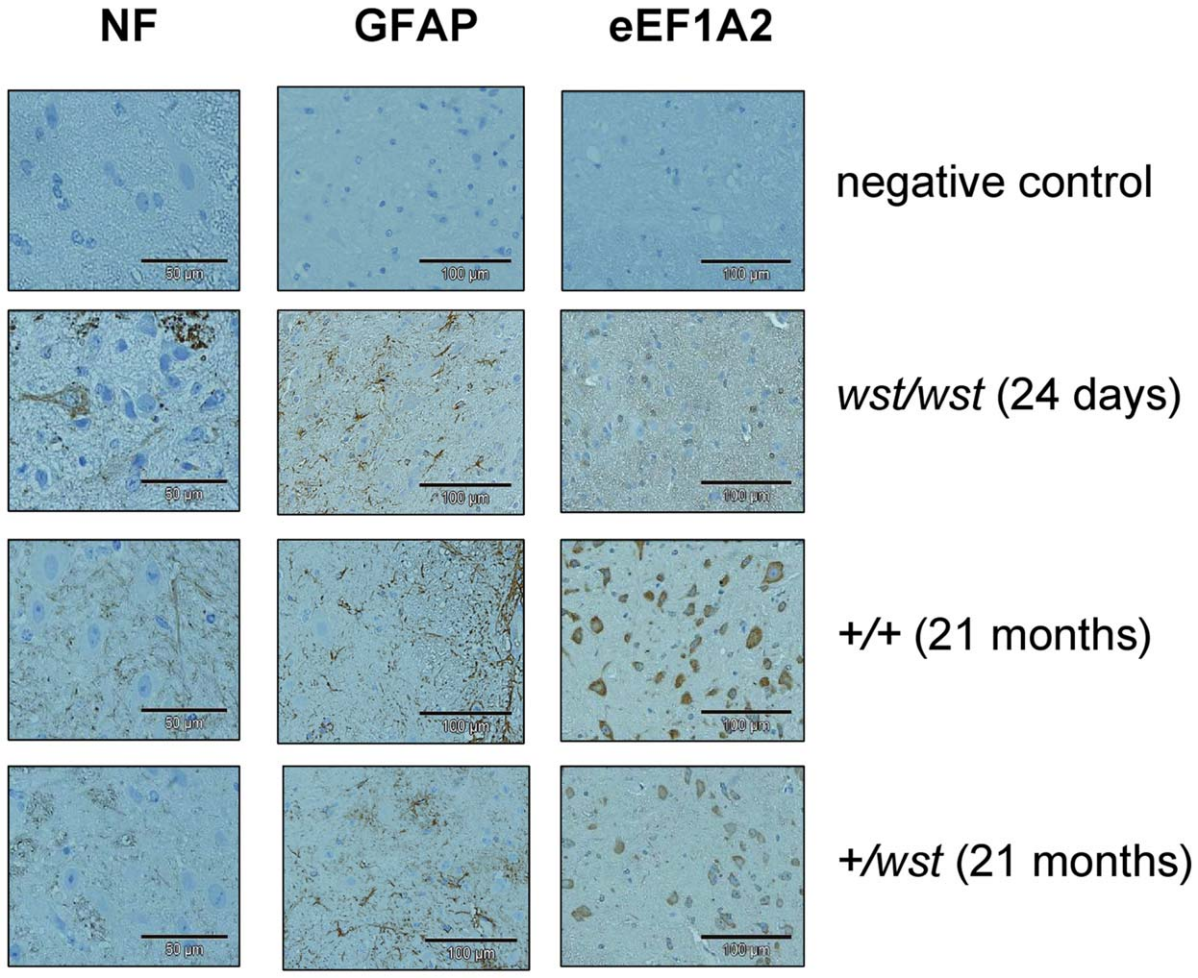
Immunohistochemistry in aged spinal cord sections. Expression of phosphorylated neurofilaments, GFAP, and eEF1A2 in cervical spinal cord sections from 21 month old mice. The top panel in each case shows a section with primary antibody omitted from the protocol, the second panel from the top shows sections from a 24 day old wasted homozygote as a control, and the bottom two panels show sections from an aged matched wild-type and heterozygous male. The NF staining clearly shows perikaryal accumulation of NF staining in the wasted mouse section but not in the aged *+/wst* mouse. The GFAP staining shows a characteristic pattern of reactive astrocytes throughout the grey matter of the spinal cord, even in the aged wild-type mouse. The eEF1A2 shows no staining at all in the section from the *wst/wst* mouse as expected, and fainter but easily detectable, albeit reduced, staining in the aged *+/wst* sample.

## Results

### Mice

A cohort of ageing male and female *+/wst* heterozygous mice together with male and female wild-type controls was established. Four groups of 8 animals were bred from 3 breeding pairs. Mice were selected from the first two litters of each breeding pair, thus there were slight differences in age of some of the litters but this was no greater than 1 month; all tests were conducted when the youngest mice were of the required age. The entire cohort was tested on the same day and all tests were carried out in the morning. Motor function was assessed using an accelerating rotarod and muscle function using a grip strength meter. Initially these animals were tested at tri-monthly intervals to allow us to pinpoint the starting time of any difference between wild-types and heterozygotes. A subsequent cohort was aged to 18 months consisting of 3 wild-type males, 3 heterozygous males, 3 wild-type females and 4 heterozygote females with no testing until this age.

**Figure 5:**
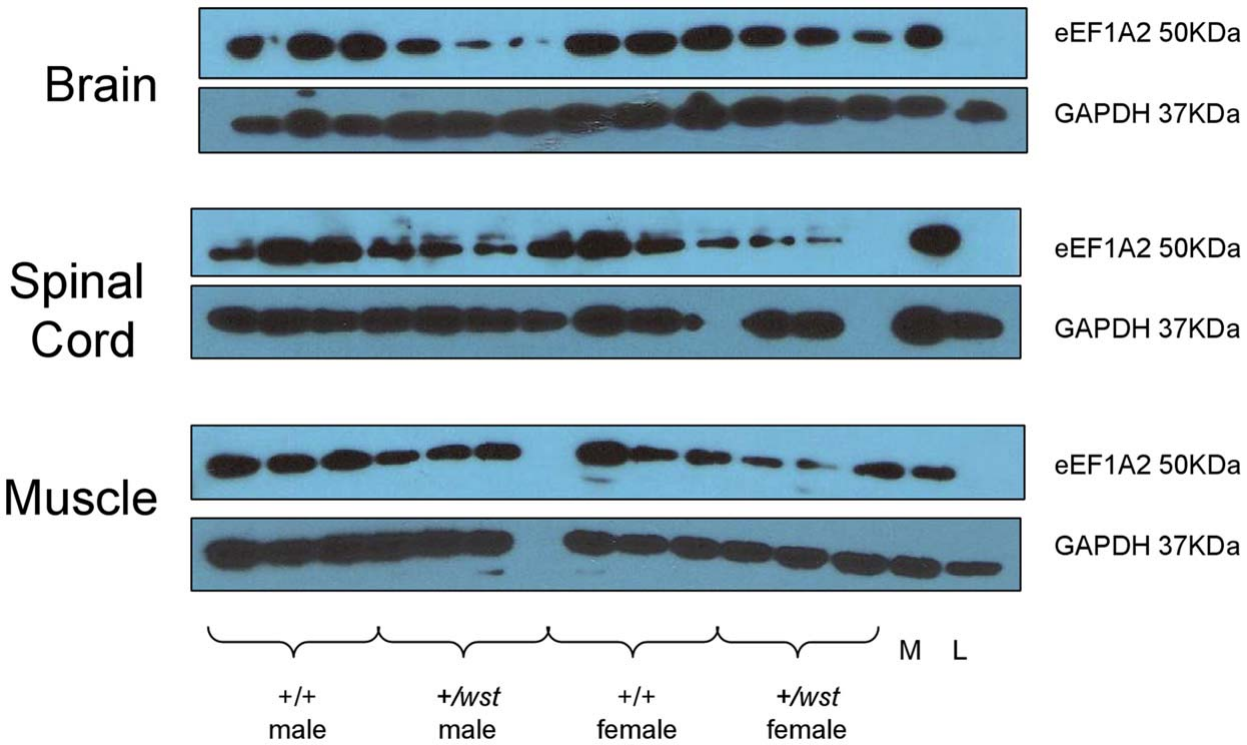
Protein expression in aged mice. Western blots showing protein expression of eEF1A2 and GAPDH (as a loading control) of animals from the ageing cohort. Three 21month old animals from each group are shown. WT indicates wild-type animals, HET indicates heterozygous animals, M indicates a muscle sample (as an eEF1A2 positive control) and L indicates a liver sample (an eEF1A2 negative control), both from wild-type mice.

### No Differences in Rotarod Performance

Rotarod performance was analysed at tri-monthly time points until 15 months of age, and then more frequently until 21 months. As males and females perform differently in some behavioural tasks such as the rotarod, data were analysed by genotype and also by gender. The mean monthly fall times for each group are shown in Figure 1. No significant differences were observed between wild-types and heterozygotes of either gender between the ages of 3 and 21 months of age. The data were also analysed by normalising the mean times to body weight (data not shown) but again, no significant difference was found between groups. The performance of all four groups declined over the first 15 months, but then improved when monthly testing was introduced.

### Grip Strength is Sensitive to Total Loss of eEF1A2 but not Haploinsufficiency

The results of the grip strength assay in wasted homozygous mice and their normal littermates (both heterozygous and wild-type mice) show a significant difference between wasted mice and controls from 20 days in both the forelimbs only and in all four limbs (Figure 2). Oddly, for both sets of measurements, the difference between wasted and control mice becomes at best weakly significant at 21 days but returns to being highly significant by 22 days. From 20 days onwards, at least 8 mice were tested in each category. These results validate the grip strength meter as a useful test for distinguishing wasted mice from their littermate controls.

Having shown that this assay successfully distinguished *wst/wst* mice from normal littermate controls, we went on to use it to measure muscle function in heterozygotes and wild-type littermate controls. Mice were tested tri-monthly from 3 months of age to 21 months of age. Readings were taken from forelimbs only and from all four limbs combined. On a single test day, three readings were taken for each assay. In any given testing month these tests were carried out two days after the last rotarod test day.

Figure 3 shows the grip strength test results for the whole testing period. Mean data for each animal were normalised to body weight before combining the group data. There was no statistically significant difference between any of the groups at any time point over the course of the study. Until the mice reach 15 months, age appears to have no effect on performance, but between 16–18 months there appears to be an increase in performance in all groups. This is most likely due to the increase in exercise the animals received during this time period when the frequency of rotarod testing was increased.

### No Significant Pathology in Heterozygous Mice

Once the rotarod and grip strength experiments were terminated, spinal cords were collected from the mice and analysed for potential markers of motor neuron degeneration. Sections were initially stained with an antibody that recognises phosphorylated neurofilaments (both light and heavy chain). These proteins typically accumulate in the perikarya of neurons in cases of motor neuron degeneration; this effect is seen quite markedly in homozygous *wst/wst* mice by 19 days [2]. No significant differences were observed between wild-type and heterozygous animals, although clear perikaryal staining can be seen in a motor neuron on the control section from a wasted homozygous mouse (Figure 4, left hand panel and Figure S1).

Sections were also stained with an antibody for glial fibrillary acidic protein (GFAP) as a marker of gliosis. In wasted mice reactive gliosis is the earliest observable abnormality in the spinal cord [2]. Although there were indications of reactive gliosis, as characterised by numerous GFAP positive foci in the grey matter of the spinal cord, no differences were observed in GFAP staining between wild-types and heterozygotes (Figure 4, central panel and Figure S1). As wasted mice display a rostrocaudal gradient of gliosis, with the cervical region being more affected than the thoracic and lumbar levels, all regions of the spinal cord were examined in this study. However, no difference was seen at any level of the spinal cord (data not shown).

Spinal cord sections were also analysed for expression of eEF1A2, which was also used to stain motor neurons for counting. Clear motor neuron-specific staining was seen, albeit at a slightly reduced level in *+/wst* mice (Figure 4, right hand panel).

Finally, counts of motor neurons were conducted on the spinal cord sections. No sign of vacuolation was seen in any case, and there were no statistically significant differences in MN counts between *+/wst* and *+/+* male mice (Table 1), although the numbers counted on different sections were highly variable. No differences were seen in female mice either, but data are not shown as only two female heterozygotes were available.

### Expression Analysis of eEF1A2

As no pathological differences could be seen between wild-type and heterozygous mice, we measured protein levels of eEF1A2 in brain, spinal cord and muscle by Western blotting in order to establish whether levels of eEF1A2 in heterozygotes were really reduced to the predicted 50%, or whether some compensatory mechanism had caused them to be increased closer to wild-type levels. Three individuals from each of the four groups were analysed at 21 months of age (Figure 5). Expression of eEF1A2 does indeed appear to be decreased in heterozygote animals relative to wild-type (both males and females) in all three tissue types, but results were very variable, in part because of the lower sample number caused by reduced survival in the aged animals, and because there is clear variability between individual animals. Analysis of further aged mice showed the same very variable decrease in expression in aged heterozygotes (data not shown). This analysis did establish, however, that there had not been a compensatory increase in eEF1A2 in heterozygous mice that could have accounted for the absence of phenotypic abnormalities in the aged heterozygotes. Indeed, it is apparent from these results that levels of substantially less than 50% of those seen in wild-type mice (only 7.5% in male heterozygous brains, for example) is compatible with normal neuromuscular function.

## Discussion

Total ablation of eEF1A2, as seen in wasted homozygous mice, gives rise to an early onset and extremely aggressive phenotype resulting in death by 4 weeks of age. This, together with the fundamental housekeeping role of eEF1A2 in protein synthesis, suggested that a decrease in eEF1A2 levels beyond a certain threshold could compromise the function of motor neurons and/or muscle. We therefore aged and analysed a cohort of heterozygous wasted mice, presumed to have 50% of wild-type expression levels.

Initially, we tested heterozygous and wild-type littermate mice on the rotarod at a variety of ages, to assess gross motor function and coordination. In these studies both wild-type and heterozygous animals demonstrated improvement with practice in the rotarod test, which could be attributed either to learning or to beneficial effects of regular exercise. This is shown most clearly when only one month elapsed between tests; here, performance in all groups increased compared to performance in tests conducted every three months. This is consistent with results obtained on similar assays conducted on mice carrying the SOD1 G93A mutation [13], with both wild-type controls and transgenic mice showed evidence of learning. A further study demonstrated the beneficial effects of exercise in the same line [14], with regular treadmill exercise over a 10 week period significantly extending the lifespan of the diseased animals. Another group showed that exercise delayed the onset of disease in females but not in males and had no affect on lifespan of either sex [15]. It is conceivable, therefore, that regular exercise could mask any potential difference between wild-types and heterozygous mice in this study. However, the follow-up study, albeit on restricted numbers of animals, also failed to show any difference between cohorts of different genotypes even though the animals were not tested until 18 months of age. This suggests that there is genuinely no difference in performance of wild-type and heterozygous mice. This conclusion is supported by the grip strength analysis which, although it proved to be a robust assay for distinguishing wasted mice from their littermate controls, also failed to show any difference between the groups of different genotype in the aged cohort.

Further, we found no significant differences in terms of spinal cord pathology between any of the groups examined using GFAP and NFH as markers of neurodegeneration. It has been demonstrated that levels of GFAP in the mouse central nervous system increase with age, at least in the brain [16], [17], [18]. This may explain the high level of staining in older wild-types that complicated our analysis; we are not aware of any studies of GFAP in ageing spinal cords. Motor neuron counts also demonstrated no significant difference between groups. However, animals were being lost for unrelated health reasons (similar numbers of heterozygotes and wild-type mice) by this stage, so that relatively few animals were available at the endpoint of the study. Since the results also showed substantial variation between mice, this aspect of the work could be regarded as inconclusive.

Expression analysis of eEF1A2 in the heterozygotes at the end stage of this study showed that, depending on the sex of the animals and the tissue studied, eEF1A2 levels in heterozygotes relative to their wild-type littermates decline markedly with age when compared to the results at four weeks published by Khalyfa et al [19]. Recent results in other tissues such as heart and hippocampus have concluded that only minor changes occur in the mouse proteome as a whole during aging [20], but as this study did not examine skeletal muscle it is impossible to judge whether our result is a feature of muscle, or is protein-specific.

It is interesting to compare our results on eEF1A2 with those found for *Zpr1* knockout mice. In this case, heterozygous *Zpr1+/−* mice do show some reduced expression when compared to wild-type controls, albeit less than predicted [9], and this reduction in level becomes more marked with age. However, in contrast to eEF1A2 where levels are found at the predicted 50% of wild-type in younger heterozygous mice [19], ZPR1 levels in the brains of heterozygous *Zpr1+/−* knockout mice start at almost 90% of wild-type levels, declining to 30% by 12 months of age. Whilst 6 week old heterozygotes showed no marked decrease in motor neuron number compared to wild type controls, by 12 months of age heterozygous mice display reduced cell density and loss of anterior horn motor neurons compared to wild-types. This correlates directly with the decrease in ZPR1 protein with age. ZPR1 has been shown to preferentially bind to GDP bound eEF1A [21]. As eEF1A2 has a slower disassociation rate than eEF1A1 it is possible that ZPR1 preferentially binds eEF1A2. It is therefore plausible that in ageing wasted heterozygotes the reduced levels of eEF1A2 result in insufficient ZPR1-eEF1A complexes. An interesting avenue for future work would be to examine whether any changes occur in the levels or localization of ZPR1 and SMN in older heterozygous *+/wst* animals, which would contribute to further our understanding of the interaction between these three proteins. Crosses between *Zpr1+/−* and *Eef1a2+/−* mice could also be carried out, to establish whether decreased levels of both eEF1A2 and ZPR1 can compromise motor neuron function further.

## Supporting Information

Figure S1 Higher resolution images of GFAP and NF-H staining in aged heterozygous and wild-type mice.
(TIF)
