# Supplementary figures and images for: Haploinsufficiency for Translation Elongation Factor eEF1A2 in Aged Mouse Muscle and Neurons Is Compatible with Normal Function
Source: PLoS One. 2012 Jul 25;7(7):e41917. doi: 10.1371/journal.pone.0041917 (PMC3405021; doi:10.1371/journal.pone.0041917)

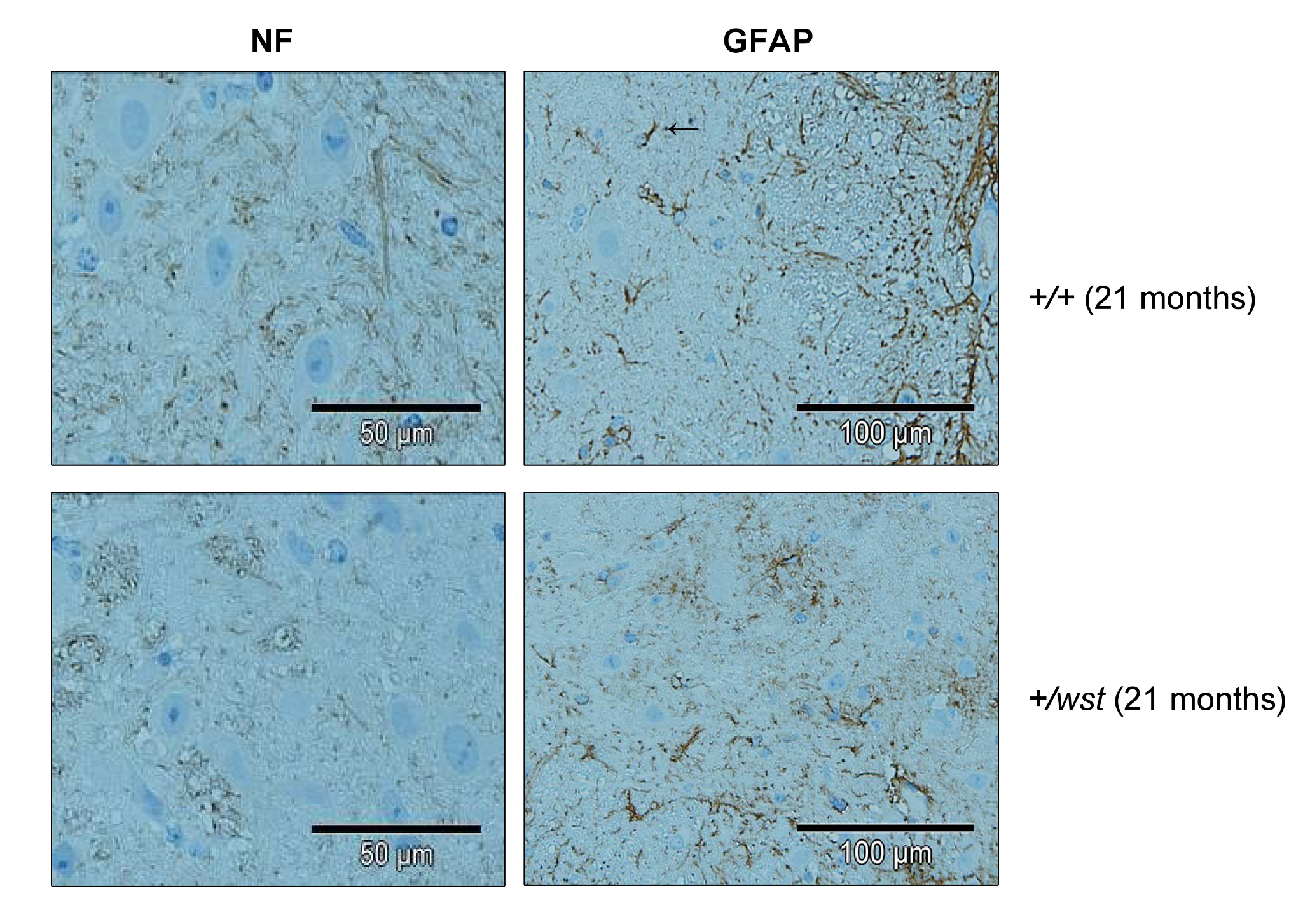

Supplement: Figure S1 — Higher resolution images of GFAP and NF-H staining in aged heterozygous and wild-type mice. (TIF) [file pone.0041917.s001.tif]
